# Characteristics of a novel cell line ZJU-0430 established from human gallbladder carcinoma

**DOI:** 10.1186/s12935-019-0911-1

**Published:** 2019-07-22

**Authors:** Fei Zhou, Yanhua Zhang, Jihong Sun, Xiaoming Yang

**Affiliations:** 1 Department of Radiology, Sir Run Run Shaw Hospital, Zhejiang University School of Medicine, Hangzhou, Zhejiang China; 2 Department of Pathology, Sir Run Run Shaw Hospital, Zhejiang University School of Medicine, Hangzhou, Zhejiang China; 3 Image-Guided Bio-Molecular Intervention Research, Department of Radiology, University of Washington School of Medicine, Seattle, WA USA

**Keywords:** Gallbladder cancer, ZJU-0430 cell line, Short tandem repeat, Epithelial, Karyotype analysis, Tumorigenicity

## Abstract

**Background:**

Gallbladder cancer is the most common malignant neoplasm of the biliary tract, responsible for 80–95% of cases. Appropriate models are required for investigating the molecular pathogenesis of gallbladder cancer.

**Methods:**

In this study, we aimed to establish a gallbladder cancer cell line from primary tumour. Single cell RNA sequencing, Light and electron microscopy, DNA content analysis, cytogenetic analysis, short tandem repeat (STR) DNA fingerprint analysis, immunophenotypic characterization, and xeno-transplantation were utilized to characterize the novel ZJU-0430 cell line in vitro and in vivo.

**Results:**

The cell line showed multiple cell shapes and characteristic epithelial morphologies under the microscope, but no too much heterogeneity by scRNA-Seq, with a population doubling time (PDT) of 19.81 h, which was shorter than that for GBC-SD cells. An immunophenotypic analysis revealed that ZJU-0430 cells were positive for CD24, CD44, CD29 and CD133 expression, and partially positive for CD184, and CD326 expression, and negative for CD34, CD90, CD117, and CD338 expression, similar to the primary cancer cells. A pathological analysis confirmed the origination of cell line from gallbladder tumour. ZJU-0430 cells had higher migration, invasion and proliferation properties than GBC-SD cells in vitro, and showed in vivo tumorigenicity in nude mouse xenograft settings.

**Conclusions:**

The results confirm the potential utility of ZJU-0430 cell line as a representative model of gallbladder cancer and suggest that it could be used in the in vitro and in vivo studies of gallbladder cancer pathogenesis and to develop new therapeutics.

**Electronic supplementary material:**

The online version of this article (10.1186/s12935-019-0911-1) contains supplementary material, which is available to authorized users.

## Background

Gallbladder carcinoma (GBC) is an invasive adenocarcinoma that originates from the epithelial linking of the digestive system [1]. GBC is a common aggressive malignant neoplasm and the fifth most deadly cancer, initiating from the gallbladder or cystic duct. Chronic cholecystitis (CC) with gallstones, dietary factors, chronic gallbladder infections, and environmental exposure to specific chemicals are considered as main risk factors for the development of GBC [2], which has a wide incidence worldwide [3–5].

Despite the progress in therapeutic strategies, the overall survival rate has remained poor, mainly due to late diagnosis, early metastasis, ineffective surgical resection, and insensitivity to chemoradiation [1, 6–8]. Therefore, it is essential to further investigate its biological behaviours, mechanisms, and potential treatments. In recent years, cancer cell lines originating from patients have proven to be a powerful tool that can be used for drug screening, drug resistance research, analysis of the tumour microenvironment, gallbladder cancer pathogenesis and the mechanism of metastasis [9, 10]. Previously, only a few GBC cell lines derived from primary tumours have been established but insufficiently elaborated upon [11–27]. This situation necessitates the establishment of more novel GBC cell lines for studying it in detail.

In this study, a novel gallbladder cell line derived from a primary GBC, referred to as ZJU-0430, was successfully established. All our data together confirmed that it as a potentially useful model for the further study of this disease.

## Methods

### Patient history

This study was performed in accordance with the Declaration of Helsinki of 1975, and the official recommendations of Chinese Community Guidelines, and was approved by the Ethics Committee and Institutional Review Board of the Sir Run Run Hospital. Written informed consent was obtained from the patient.

A 74-year-old male patient with pain in the upper abdomen was admitted in our centre. Gastroscopy showed the presence of a gastric antral ulcer (stage S2), and irregular deep concave ulcer at the gastric angle, and cancer priority. Abdominal computed tomography (CT) also found a 1.5-cm thick wall at the bottom of the gallbladder. A radioimmunoassay showed that the patient’s serum levels of a variety of biomarkers were normal (CA19-9, CA-125, AFP, CEA, and PSA) except for ferritin, which was high (409.3 ng/ml, 30–400 ng/ml). A radical resection of stomach and gallbladder were performed and a pathological examination showed that gastric carcinoma was a poorly differentiated adenocarcinoma derived from signet-ring cell carcinoma, whereas the primary gallbladder cancer was a well differentiated adenocarcinoma.

### Cell lines as control

GBC-SD cells were obtained from the Cell Bank of Type Culture Collection of Chinese Academy of Sciences (Shanghai, China), and maintained in Roswell Park Memorial Institute (RPMI) 1640 medium (Invitrogen, Carlsbad CA, USA) supplemented by 10% fetal bovine serum (FBS) (Gibco, Grand Island, NY, USA), and 1% penicillin/streptomycin and amphotericin B (Invitrogen).

### Cell culture

The approach of primary culture was as previously described [28]. Single-cell suspensions were obtained according to the manufacturer’s protocol (Miltenyi Biotec GmbH, Germany). Specifically, the surgically resected gallbladder specimens were immediately transferred to the lab, rinsed several times in cold Dulbecco’s Phosphate Buffered Saline (DPBS) containing 1% penicillin/streptomycin and amphotericin B, cut into small pieces (1 mm^3^), and transferred into a gentleMACS™ C tube containing the buffer from the Tumor Dissociation Kit (human), and subjected to a gentleMACS™ Tissue Dissociator. Cells were cultured in complete growth RPMI 1640 medium (containing additives) at 37 °C in a humidified incubator containing an atmosphere of 5% CO_2_. Fibroblasts were removed by differential trypsinisation as described previously [29, 30]. Serial passages were carried out every 3–4 days, routinely at a ratio of 1:2, and the medium was replaced when colour changed. The ZJU-0430 cell line was cultured for > 100 passages and showed no changes in its morphology.

### Single cell RNA sequencing

ZJU-0430 cells were grown in mycoplasma-free complete growth RPMI 1640 medium without antibiotics. Cells were grown to 60% confluence, and washed with 4 ml of DPBS, and then treated with StemPro™ Accutase™ Cell Dissociation Reagent (Invitrogen). Cells were then centrifuged at 300 rcf for 30 s at room temperature and the supernatant removed without disturbing the cell pellet. Next, 0.04% bovine serum albumin fraction V (BSA) solution (Sigma-Aldrich, St. Louis, MO, USA) was added and then centrifugation at 300 rcf for 5 min. The resuspended cells were then filtered through 40 µm cell strainer (Corning Incorporated, Corning, NY, USA) and following Trypan Blue staining, the cell density and viability were determined using haemocytometer.

Single-cell RNA-seq libraries were prepared with Chromium Single cell 3′ Reagent v2 (or v3) Kits according to the manufacturer’s protocol. Single-cell suspensions were loaded on the Chromium Single Cell Controller Instrument (10 × Genomics) to generate single cell gel beads in emulsions (GEMs). In order to do this, ZJU-0430 single cells were suspended in DPBS containing 0.04% weight/volume BSA. About cells were added to each channel with a targeted cell recovery estimate of cells. After generation of GEMs, reverse transcription reactions were performed to generate barcoded full-length cDNA followed by the disruption of emulsions using the recovery agent and cDNA clean up with DynaBeads Myone Silane Beads (Thermo Fisher Scientific, MA, USA). The cDNA was then amplified by PCR with appropriate an appropriate number cycles which depending on the recovery cells. Subsequently, the amplified cDNA was fragmented, end-repaired, A-tailed, index adaptor ligated and library amplification. These libraries were sequenced on the Illumina sequencing platform (HiSeq X Ten) and 150 bp paired-end reads were generated.

The Cell Ranger software pipeline (version 2.2.0) provided by 10 × Genomics was used to demultiplex cellular barcodes, map reads to the genome and transcriptome using the STAR aligner, and down-sample reads as required to generate normalized aggregate data across samples, producing a matrix of gene counts versus cells. We processed the unique molecular identifier (UMI) count matrix using the R package Seurat (version 2.3.4). To remove low quality cells, we filtered those cells which with UMI larger than 8000 and gene lower than 2000. And also we discarded low-quality cells where > 20% of the counts belonged to mitochondrial genes. After applying these QC criteria, 11,083 single cells and 20,118 genes in total remained and were included in downstream analyses. Library size normalization was performed in Seurat on the filtered matrix to obtain the normalized count. Top variable genes across single cells were identified using the method described in Macosko et al. [31]. Principal component analysis (PCA) was performed to reduce the dimensionality on the log transformed gene-barcode matrices of top variable genes. Cells were clustered based on a graph-based clustering approach, and were visualized in 2-dimension using tSNE and UMAP. Likelihood ratio test that simultaneously test for changes in mean expression and in the percentage of expressed cells was used to identify significantly differentially expressed genes between clusters. Enriched biological function were analysis with DAVID website and fgsea packages in R.

### Tumorigenicity

All BALB/c nude mice experiments were carried out in accordance with Animal Research: Reporting of In Vivo Experiments (ARRIVE) guidelines, and were approved by the Zhejiang University Animal Care and Use Committee (ZUACUC) (Project License 11627). To determine the oncogenicity of the ZJU-0430 cell line, cells were subcutaneously injected into the hind flanks of four nude mice (BALB/c nu; 4–6 weeks old) (SLAC Laboratory Animals Company, Shanghai, China), Mice were maintained in laminar flow cabinets under specific pathogen-free conditions. A total of 1 × 10^6^ cells were injected using a 27-gauge needle and the tumours monitored at intervals using digital callipers. After 3 weeks, mice was killed by cervical dislocation under sodium pentobarbital (45 mg/kg) anaesthesia. Tumours were removed, fixed in 10% formalin and subjected to pathological examination and IHC evaluation.

### Light and electron microscopic analysis

Both the morphology and ultrastructure of the ZJU-0430 cells were examined following seeding into 25-mm^3^ tissue-culture flasks. The general morphology of the cells was observed daily under a phase-contrast microscope and histopathologically compared to that of the original tumours. Electron microscopy was conducted as previously described [32] using the GBC-SD cell line as the control. Cells were harvested, pelleted, fixed (2.5% glutaraldehyde), post-fixing (2% osmium tetraoxide) and then embedded in EPON resin. Ultrathin sections were stained and examined under a HT7700 microscope (Hitachi, Tokyo, Japan).

### In vitro growth kinetics and cell cycle analysis

The cell growth curve analysis was obtained, as described previously [33]. The ZJU-0430 cells and GBC-SD cells were plated at 5000 cells per well, cultured at 12 h intervals for 3 consecutive days, and cell growth determined using an MTT assay, respectively. The growth curve was plotted, and doubling time was calculated as described (http://www.doubling-time.com/index.php).

ZJU-0430 and GBC-SD cells were plated, harvested, washed twice with cold DPBS, and fixed overnight in 70% ethanol at 4 °C, treated for 30 min with RNase A (2 μg/ml), and then with propidium iodide (PI) at room temperature at the dark. The cell cycle distribution was determined using a FACSCanto™ II flow cytometer (Becton–Dickinson, Mount View, VA, USA) and analysed using appropriate software.

### Cytogenetic analysis and STR genotyping

Cells at the primary and 100th passage were used for analysis. Karyotyping was performed as described previously [34]. Briefly, cells were treated with 0.02 μg/ml Colcemid for 90 min at 37 °C until exponential proliferation was reached. The cell pellet was resuspended with potassium chloride (0.075 mol/l), incubated for 20 min at 37 °C, fixed in Carnoy’s solution (methanol: acetic acid = 3:1 by volume), and analyzed using trypsin G banding.

To verify that ZJU-0430 cell line was indeed derived from humans and had no-contamination, genomic DNA from ZJU-0430 cell lines was extracted using the Gentra Puregene Cell Kit (Qiagen, Inc., Valencia, CA) according to the manufacturer’s protocol. STR profiling was performed by amplifying eighteen loci simultaneously in a single tube and analyzing by capillary electrophoresis on an ABI Prism^®^ 3500 × 1 Genetic Analyzer. Data were matched with the American Type Culture Collection (ATCC) STR database (ATCC sales order number: SO0146233).

### Test for mycoplasma contamination

The cell-culture supernatant was collected and evaluated using the PCR Mycoplasma Test Kit (HuaAn Biotechnology, Hangzhou, Zhejiang, China), following the manufacturer’s instructions. PCR products were separated by electrophoresis in 1% agarose and documented by photography.

### Immunophenotypic characterization of ZJU-0430 cells by flow cytometry

The primary and 100-passage cells were collected for flow cytometry analysis using a FACSCanto™ II flow cytometer (Becton–Dickinson, Mount View, VA, USA), respectively. The panel of monoclonal antibodies used included those specific for CD24, CD44, CD29, CD34, CD90, CD117, CD133, CD184, CD326, and CD338. All antibodies and reagents were purchased from BioLegend (San Diego, CA). Data were obtained using FCS Express Software (De Novo Software, Los Angeles, CA). Antigen expression was scored as positive based on a significant shift in staining in comparison to corresponding isotype control.

### Wound-healing assays

This procedure was conducted by the Cell Comb™ Scratch Assay according to the manufacturer’s instructions (Merck, Darmstadt, Germany). Briefly, a wound was created in the cell monolayer using cell comb, when the cells reached semi-confluence, and they were cultured thereafter for an additional 72 h at 37 °C and 5% CO_2_. Eight scratched fields were randomly chosen for cell counting.

### Migration and invasion assays

The in vitro migration and invasion assays were performed as previously described [35, 36]. Briefly, 1 × 10^4^ cells were seeded into cell-culture insert wells (Corning Incorporated, Corning, NY, USA) with or without Matrigel-coating (BD Biosciences, Bedford, MA, USA) in RPMI 1640 medium. The extent of migration and invasion was determined after cultured for 48 h. The cells on the lower surface were fixed and stained with Giemsa solution.

### Colony formation

A colony formation assay was used to assess the proliferative ability of the ZJU-0430 cells. A single-cell suspension was prepared and plated on 6-well plates (200 cells/well) in triplicate and cultured for 14 days. The colonies were stained with 1% crystal violet for 30 s after fixation with 4% paraformaldehyde for 5 min.

### Immunostaining

Tissue section slides from original patient (GBC and gastric cancer), ZJU-0430 tumours grown in mice, and cytospins of ZJU-0430 cells were used in this experiment as described previously [37, 38]. For cytospins, ZJU-0430 cells were plated onto glass coverslips, fixed with 4% paraformaldehyde (PFA), blocked with 10% goat serum, incubated with primary antibodies (vimentin, CK-pan, CK7, and CK19 all from cell signaling technology (CST)) at 4 °C overnight, followed by the addition of immunofluorescent labelled antibodies [FITC/PE goat anti-rabbit IgG (H + L)]. For tissue slides, 5 μm sections were fixed onto the slides, dewaxed, rehydrated, heated for antigen retrieval, incubated in 3% hydrogen peroxide to inactivate endogenous peroxidase, blocked with 5% BSA, and then incubated with primary antibodies specific for the following proteins, MUC1, MUC2, MUC4, MUC5AC, MUC6, AFP, Hepatocyte, Glypican-3, GS, CK20, CAD17, CDX2, beta-catenin, STAB 2, vimentin, MMP-2, MMP-9, CK7, and CK19. A 3,3′ diaminobenzidine tetrahydrochloride (DAB) horseradish peroxidase colour development kit (Boster) was used for visualizing target protein and Mayer’s haematoxylin (Boster) was used for counterstaining.

## Results

### Histopathologic establishment of a new GBC cell line, ZJU-0430

**Fig. 1:**
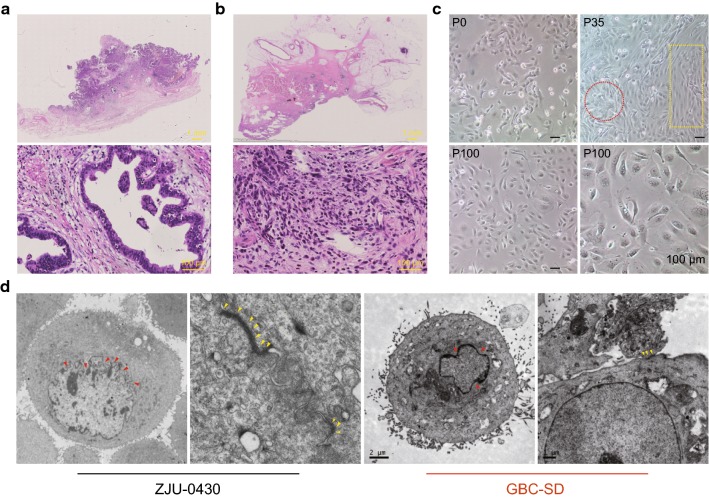
Histopathological image of original specimen and morphological characteristics of ZJU-0430 cell line. **a** H&E staining analysis of tissue from patient with GBC and from that with well-differentiated adenocarcinoma. **b** H&E staining analysis patient’s gastric cancer and from that poorly differentiated adenocarcinoma from signet-ring cell carcinoma. **c** Morphology of ZJU-0430 cell lines under a phase contrast microscope at different passages. **d** Ultrastructure features of ZJU-0430 and GBC-SD with abundants of organelle (ribosomes, rough endoplasmic reticula, mitochondria), large irregular nuclei with deep sunken (indicated by red arrow), microvillus-like projections on the cell surface (indicated by yellow arrow), and numerous desmosomes in the intercellular connections

A routine pathological examination of the original tumour was performed and compared to the similar observations made in the established cell lines. Haematoxylin and eosin (H&E) staining of the primary GBC from the patient’s surgical specimen showed well-differentiated adenocarcinoma polygonal cells that formed ribbons, nests, and sheets (Fig. 1a), characteristic of a classical GBC morphology. The gastric cancer showed a poorly differentiated adenocarcinoma derived from signet-ring cell carcinoma with clusters, beams, or scattered infiltration, with few cytoplasm, nuclear deviation, with obvious atypia and mitosis. The proliferation of interstitial fibrous tissue and capillaries is obvious (Fig. 1b). The established cell lines grew in adherent monolayer pattern with multiple forms; there was no change in the morphology during passage (Fig. 1c). Under the electron microscope, ZJU-0430 cells showed typical epithelial properties, including the presence of numerous organelles (ribosomes, rough endoplasmic reticula, mitochondria), large irregular nuclei with deep sunken caryotheca, microvillus-like projections on the cell surface, and numerous desmosomes forming intercellular connections. In contrast to ZJU-0430 cells, the GBC-SD cells had fewer microvillus-like projections on the cell surface, had sunken caryotheca, and had desmosomes that formed intercellular connections (Fig. 1d).

### H&E staining analysis and epithelial characteristics of ZJU-0430 cell line

**Fig. 2:**
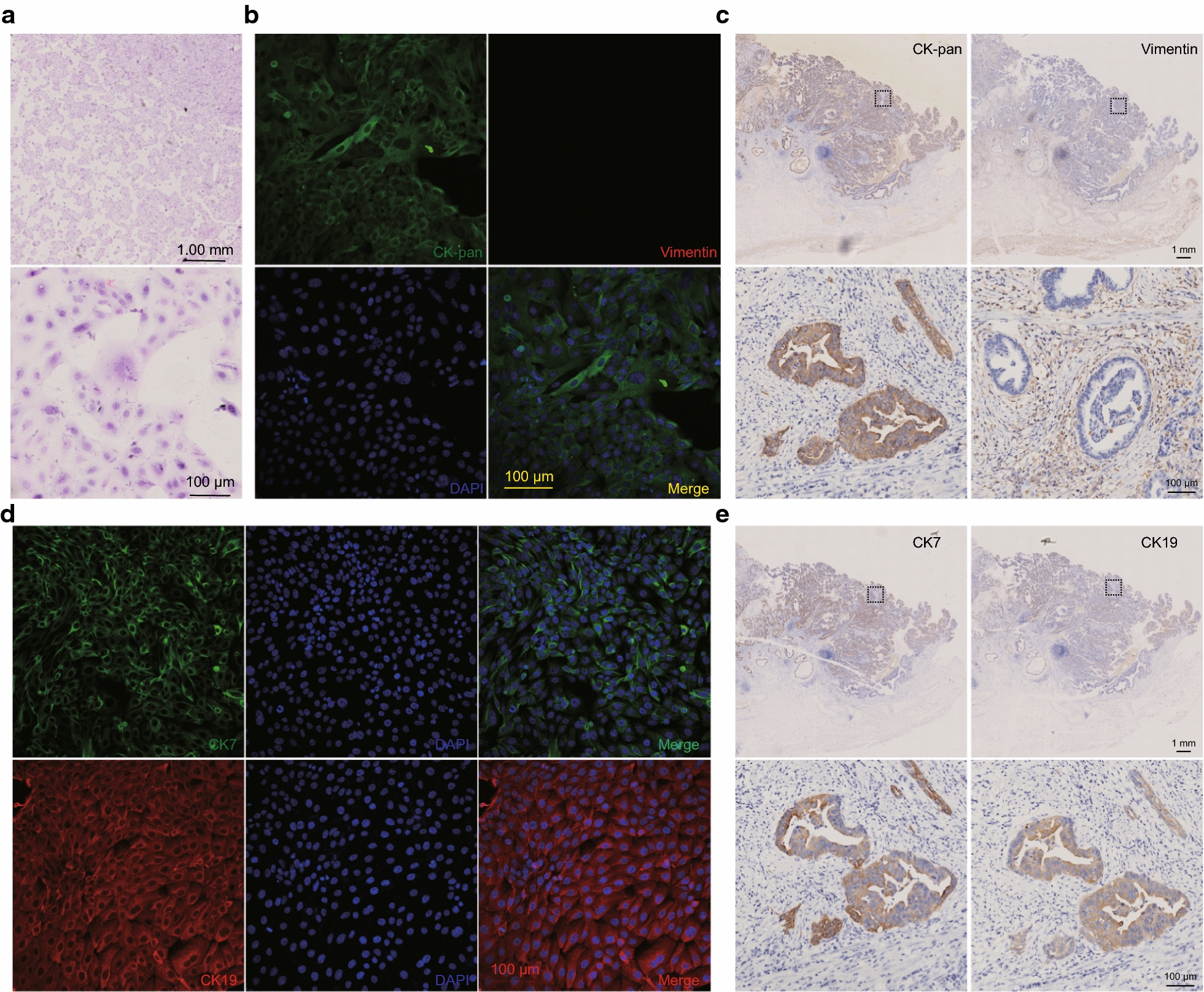
Immunostaining on slides of ZJU-0430 cells and original tumours. **a** H&E staining analysis of ZJU-0430 cells, showed moderately abundant strongly basophilic cytoplasm, ovoid nuclei with coarse chromatin and occasional irregular contours. **b**, **c** CK-pan are strongly positive in both cells and tissue sections, and vimentin is polarity. **d**, **e** CK7 and CK19 are all expressed positively on cells and tissue sections

As showed in Fig. 2a, the ZJU-0430 cell line had a moderately abundant strongly basophilic cytoplasm. The nuclei were ovoid with coarse chromatin and occasional irregular contours. The ZJU-0430 cell line and the original tumour were staining for CK-pan, vimentin, CK7, and CK19. The data showed that there was CK-pan positive staining in both the ZJU-0430 cell line and in the tumour cells in the original tissue. The ZJU-0430 cell line was negative for vimentin expression whereas the mesenchymal cells were positive for vimentin expression (Fig. 2b, c). Both the ZJU-0430 cell line, as well tumour cells in the original tissue, were positive for CK7 and CK19 (Fig. 2d, e). These results confirmed that the ZJU-0430 cell lines were originated from gallbladder epithelium.

### Mycoplasma contamination

**Fig. 3:**
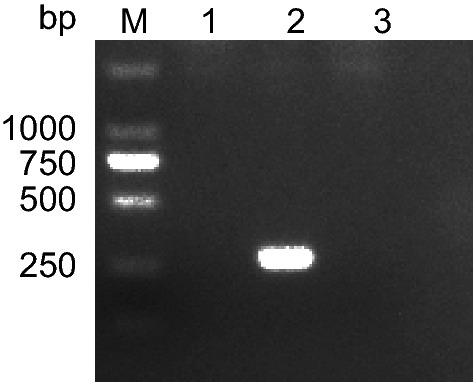
Mycoplasma examination Agarose gel electrophoresis analysis showed that the PCR products of ZJU-0430 had no relevant band (3); (1) and (2) are negative and positive controls, respectively

Uphoff and Drexler reported that 1–35% cells are prone to mycoplasma contamination in primary early passages and in continuous cell culture [39]. In order to assess if the ZJU-0430 cell line suffered from mycoplasma, mycoplasma contamination was assessed using a PCR assay. As shown in Fig. 3, no bands were detected in samples from the ZJU-0430 cell line, indicating that it is free of mycoplasma contamination.

### DNA profiles

STR profiles were prepared to confirmed the species of origin of the JU-0430 cell line. Analysis of the ZJU-0430 cell line STR profiles compare to the ATCC and DSMZ databases revealed it to be of human origin. The data also excluded the possibility of cross-contamination with other cell lines, including the HeLa cell line (Table 1, Additional files 1, 2).

**Table 1:** STR profile report of ZJU-0430 cell line

Sample of ZJU-0430 cells		
STR loci	Allele 1	Allele 2
D3S1358	17	
TH01	7	10
D21S11	30	31
D18S51	14	21
Penta_E	12	
D5S818	11	
D13S317	8	12
D7S820	11	12
D16S539	11	13
CSF1PO	11	
Penta_D	11	12
Amelogenin	X	Y
vWA	16	17
D8S1179	10	12
TPOX	11	
FGA	23	
D19S433	14	15.2
D2S1338	17	24

### Growth characterization in vitro

**Fig. 4:**
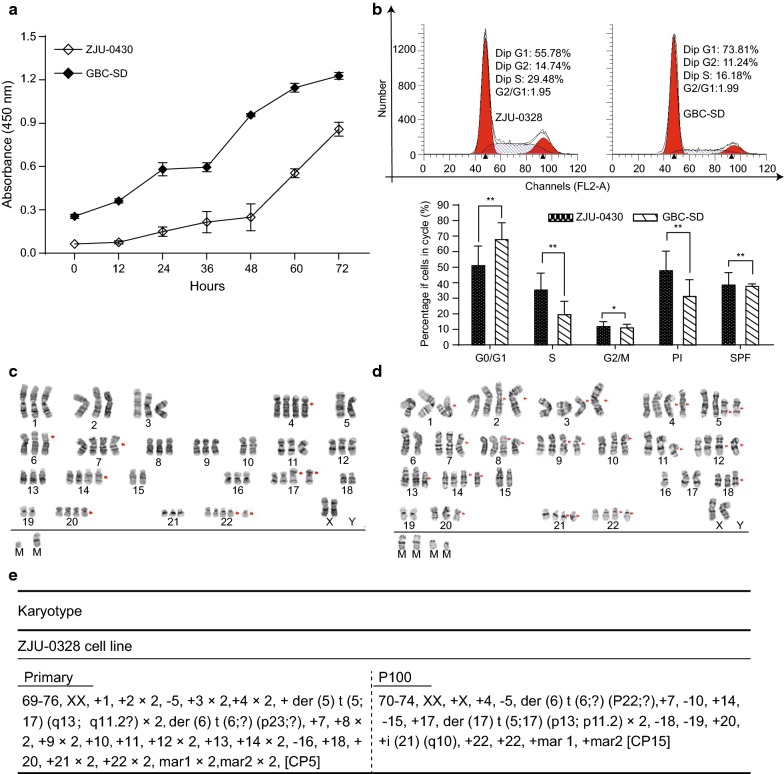
Double time, DNA content, and cytogenetic assay. **a** The PDT of ZJU-0430 cells and GBC-SD were 20 h and 33.5 h, respectively. And the growth curves are shown. **b** Representative cell cycle distribution of ZJU-0430 cells, 55.78% in G1 phase, 14.74% in G2 phase, and 29.48% in S phase, and GBC-SD cells, 73.81% in G1 phase, 11.24% in G2 phase, and 16.18% in S phase, and statistical analysis. **c**–**e** Representative G-banding profile of ZJU-0430 and GBC-SD (abnormal chromosome indicated by red box)

The ZJU-0430 cell line was characterized for its ability to proliferate and progress through the cell cycle, the GBC-SD cell lines was used as a control (Fig. 4a). The PDT of the ZJU-0430 cells and the GBC-SD cells were approximately 19.81 h and 33.5 h, respectively. The ZJU-0430 cell line grew shorter than the GBC-SD cell line on the condition of initial cell concentration.

The DNA contents of the ZJU-0430 cell line and GBC-SD cell lines were analysed by flow cytometry. ZJU-0430 cells had higher PI (**P < 0.05) and SPF (**P < 0.05) than GBC-SD cells (Fig. 4b), and therefore have a higher proliferative potential compared with the GBC-SD cells.

### Cytogenetic analysis

Chromosomal abnormalities such as number and structure abnormalities are frequently found as cancer progresses. A cytogenetic analysis of the ZJU-0430 cell line at the primary stage and P100 were performed using banding technology. The results showed both numerical and structural aberrations at different passages. A complex near-triploid clone with a modal number of chromosomes of 73 showed a representative karyotype categorized as: 70–74, XX, − X, + 4, − 5, der (6) t (6: ?) (P22; ?), + 7, − 10, + 14, − 15, + 17, der (17) t (5;17) (p13: p11.2) × 2, − 18, − 19, + 20, + I (21) (q10), +22 × 2, + mar 1, + mar 2 [CP15] (Fig. 4c, e) at P100. The karyotype of primary cells was also investigated, and the results were 69–76, XX, + 1, + 2 × 2, − 5, + 3 × 2, + 4 × 2, + der (5) t (5;17) (q13; q11.2?) × 2, der (6) t (6; ?) (p23; ?), + 7, + 8 × 2, + 9 × 2, + 10, + 11, + 12 × 2, + 13, + 14 × 2, − 16, + 18, + 20, + 21 × 2, + 22 × 2, mar1 × 2, mar2 × 2, [CP5] (Fig. 4c, e). The symbol “?” represent chromosome classification and condition are unknown. These results revealed that ZJU-0430 chromosomal abnormalities included gains, losses, translocations and other events.

### Immunophenotypic characterization

In order to investigate the stemness of the ZJU-0430 cells, an immunophenotypic analysis of the ZJU-0430 cells was performed by flow cytometry. The immunophenotypes of the primary and 100th passage cells were almost identical. The ZJU-0430 cells were positive with CD24, CD44, CD29, and CD133, partially positive for CD184, and CD326; and negative for CD34, CD90, CD117, and CD338 (Table 2, and Additional file 3: Figure S1). The result show that the ZJU-0430 cells have partial stemness.

**Table 2:** Immunophenotype profile of primary ZJU-0430 cells as determined by flow cytometry

Phenotype	Primary cells	P100 cells
CD 24	+	+
CD 44	+	+
CD 29	+	+
CD 34	−	−
CD 90	−	−
CD 117	−	−
CD 133	+	+
CD 184	±	±
CD 326	±	±
CD 338	–	–

− Negative staining, + positive staining, ± dim/partial staining

### Migration and invasion of ZJU-0430

**Fig. 5:**
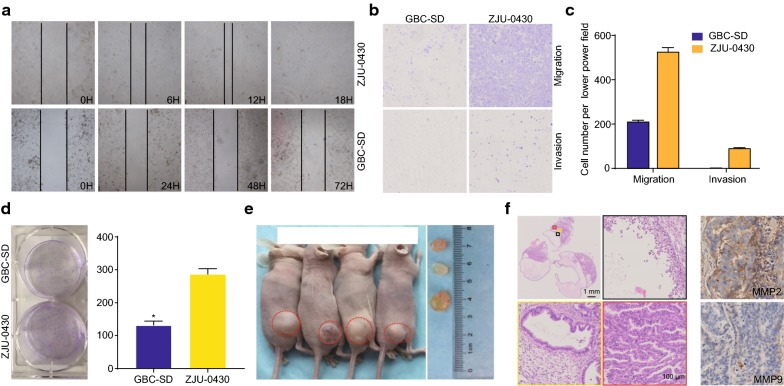
Wound healing, Transwell, colony formation in vitro, and xenograft tumours in vivo, and MMP-2, and MMP-9 protein expression. **a** Representative phase-contrast images of wound-healing assay show that migration activity of ZJU-0430 was greater than that of GBC-SD cells; **b** transwell assay (with or without Matrigel) was performed, and the number of ZJU-0430 cells that invaded the basement membrane was significantly more than that of GBC-SD; **c** ZJU-0430 cells exhibited compromised tumour formation, compared to GBC-SD cells; **d** Tumorigenicity of ZJU-0430 cells in nude mice. Tumour development was observed in both mice and gross examination found the tumour to be round or oval and often accompanied by mucus production. **e** H&E analysis showed that the atypia of tumour cells was obvious and when arranged with a small strip of cord, they formed glands as seen under the microscope. MMP-2 protein was positively expressed in the xenograft of nude mice, with negative MMP-9 protein expression

In order to investigate the ability of the ZJU-0430 cells to migrate, invade, and proliferate, wound-healing and Transwell (with or without Matrigel) assays were performed. As shown in Fig. 5a, although the scratch wound on ZJU-0430 cells was almost closed after 18 h culture, the scratch wound distance of the GBC-SD cells did not change after 72 h culture. As shown in Fig. 5b, the number of ZJU-0430 cells that invaded the basement membrane (with or without Matrigel) was significantly higher than that of GBC-SD cells.

### Colony formation

A colony formation was also performed to examine the ability of ZJU-0430 cells proliferate. As shown in Fig. 5c, ZJU-0430 cells form more compromised tumours than GBC-SD cells.

### Nude mice studies

In order to investigate their in vivo tumorigenic capacity, ZJU-0430 cell suspensions were injected subcutaneously into the flank of nude mice. Tumour development was observed in all of mice and no obvious metastatic lesions were observed. Gross examination revealed that the tumours were round or oval shaped, and often showed mucus production. The atypia of tumour cells was obvious, when arranged with a small strip of cord and viewed under a microscopy they formed different cavities that included foam cells, cancer cells and normal epithelial cells (Fig. 5d, e).

### Immunohistochemistry

**Fig. 6:**
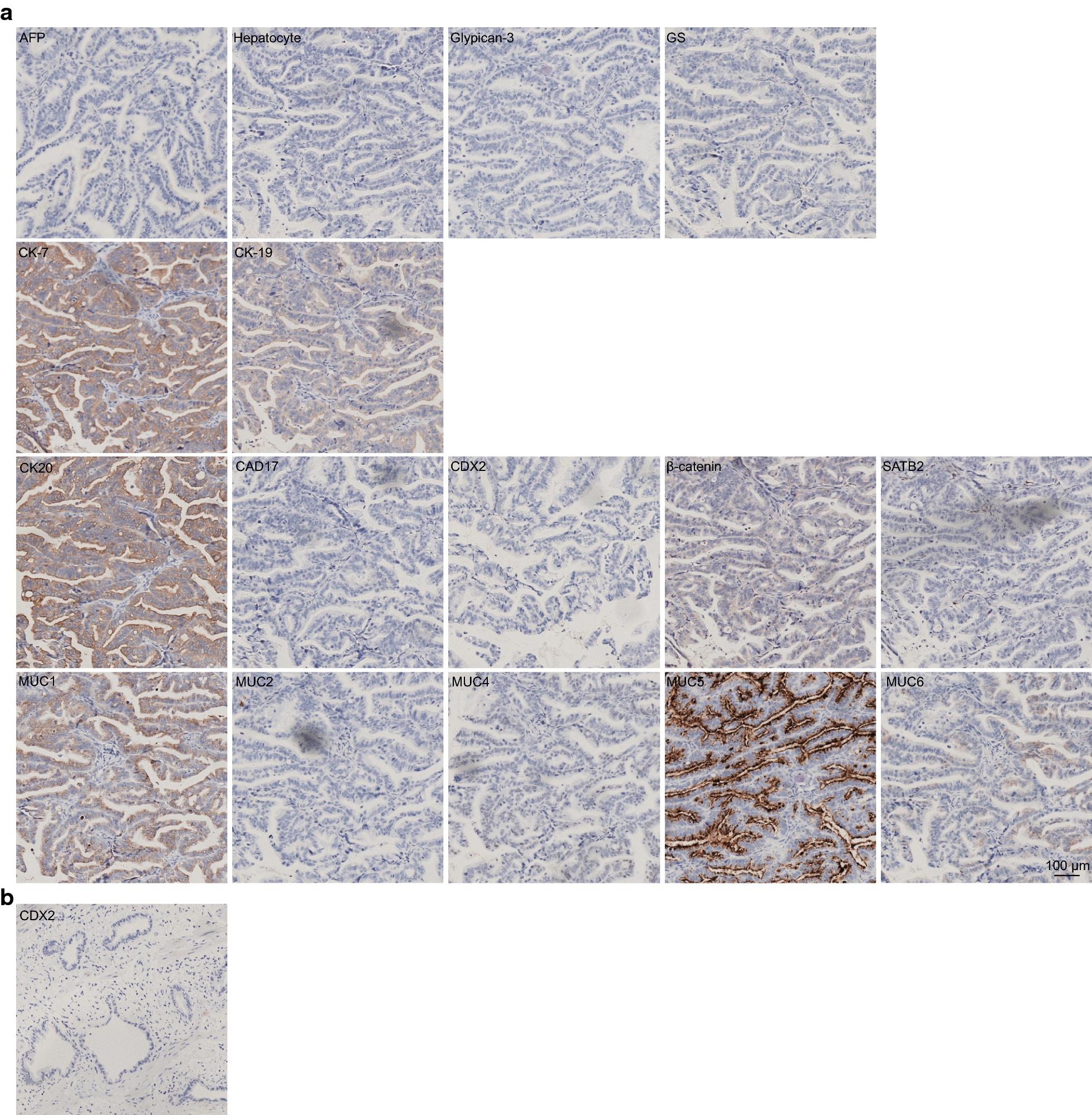
IHC for xenografted tumour sections and original GBC section. **a** ZJU-0430 xenografts of nude mice positively expressed biliary tract cancers epithelial marker CK7 and CK19, with negative stomach markers (MUC1, MUC2, MUC4, MUC5AC, MUC6), liver markers (AFP, Hepatocyte, Glypican-3, and GS), colon markers (CK20, CAD17, CDX2, β-catenin, and SATB2), and mesenchymal marker (vimentin) expression. **b** colon cancer related colon marker on the original GBC tissue was visualized by CDX2 antibody, and negatively expressed

To characterize the original ZJU-0430 cell lines, the original GBC, and the original gastric cancer, a series of markers were examined by IHC technology. As shown in Fig. 6a, ZJU-0430 xenografts of nude mice were positive for the expression of CK-pan, CK7, CK19, MUC5AC, MUC6, and MMP-2, and weakly expressed GS and CK20, there was no expression of AFP, Hepatocyte, Glypican-3, CAD17, CDX2, β-catenin, SATB2, MUC2, vimentin, and MMP-9. In addition, the original GBC tumour showed negatively staining CDX2 (Fig. 6b), and the original gastric cancer showed positive staining for CK20, CAD17, CDX2, and was negative for β-catenin and SATB2. These results confirm the ZJU-0430 cells originate from the original GBC (Additional file 4: Figure S2).

### Heterogeneity analysis by scRNA-Seq

**Fig. 7:**
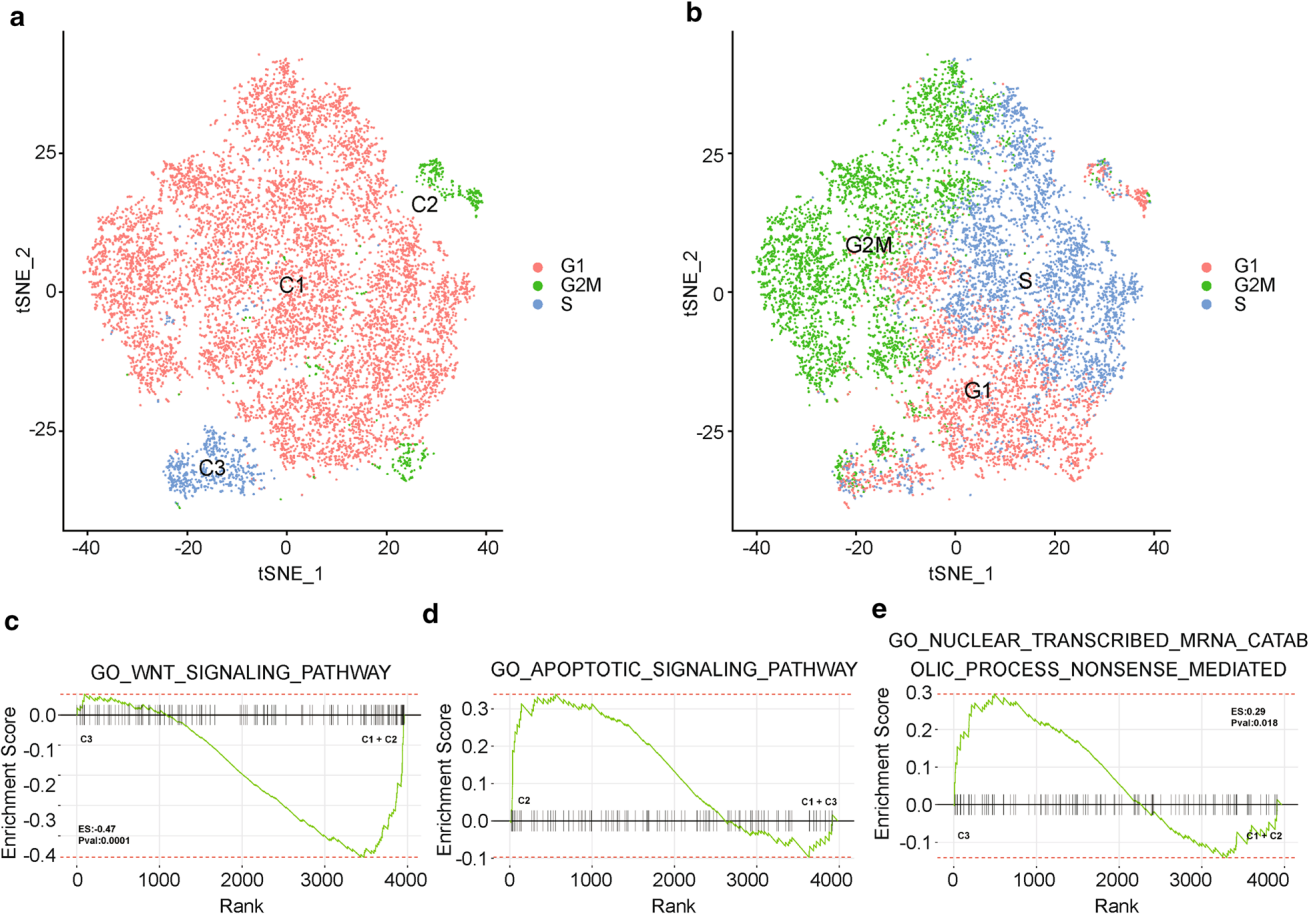
The heterogeneity of ZJU-0430 cell line by RNA-Seq. **a** tSNE plot of ZJU-0430 cell line clusters defined by Seurat pipline. **b** tSNE plot of cell cycle assignment of each cell cluster. **c** GSEA plot depicting the enrichment of genes upregulated in Wnt signaling pathway in C1 and C2 cluster. **d** GSEA plot depicting the enrichment of genes upregulated in apoptotic signaling pathway in C2 cluster. **e** GSEA plot depicting the enrichment of genes upregulated in nuclear transcribed mRNA catabolic process nonsense mediated in C3 cluster

To interpret the characteristic observed during the experimental investigation, we do single cell RNA sequencing analysis on ZJU-0430 cell line. By using Seurat pipeline, we identified 3 main cell clusters (Fig. 7a, Additional file 5: Figure S3a, b). And cell cycle effects did not contribute for the main heterogeneity as cells in different mitotic cycle presented in all 3 clusters (Fig. 7b). All those cell cluster were CD24, CD44, CD29 and CD133 positive and with partial of the cells in the C3 cluster were CD326 negative (Additional file 5: Figure S3c). To expound the characteristic of each cell cluster, we did DEG analysis between each group and using the DEGs for DAVID enrichment analysis. C1 cluster highly expressed genes associated with regulation of Wnt signaling pathway (Fig. 7c, Additional file 5: Figure S3d) and cellular component of extracellular exosome (Additional file 5: Figure S3e). C2 cluster enriched genes involved with apoptotic process (Fig. 7d, Additional file 5: Figure S3f). And C3 cluster expressed less genes and transcripts, which might due to nuclear-transcribed mRNA catabolic process associated with nonsense-mediated decay (Fig. 7e, Additional file 5: Figure S3g). These results indicated that the ZJU-0430 mainly constituted by cells with stemness signature and no too much heterogeneity.

## Discussions

Human GBC is an extremely malignant disease owning to its relative rarity, histological heterogeneity, and proximity to vital structures. Surgical resection, and chemoradiotherapy are ineffective [1, 8, 40], and the median survival is less than 10 months [41] while the 5-year survival rate is less than 5% [42]. In recent years, a series of human carcinoma GBC cell lines have been established from peritoneum effusion, surgical and autopsy tissues, biopsy specimens, and metastatic lesions [19, 26, 27, 37, 43, 44]. Novel GBC cell lines provide an ideal experiment model to investigate the biological behavior of GBC cells and may aid in the development of novel adjuvant therapies, potential anticancer agents, or new diagnostic strategies.

Cell lines derived from primary tumours are more likely to reflect the characteristics of the original primary tumour more accurately. Based on this idea, here we report the establishment of a GBC cell line designated as ZJU-0430, which was derived from a 74-year-old male patient who had some serum ferritin abnormalities, and GBC that was a well-differentiated adenocarcinoma. The cell grew in adherent monolayer pattern with at least two morphologies that co-existing. scRNA-Seq results revealed that ZJU-0430 cell line was no too much heterogeneity although the morphological difference. Optical microscopy, ultrastructural examination, immunostaining of epithelial markers for positive (CK-pan, CK7, and CK19) and negative mesenchymal expression (vimentin), and H&E staining of cells displayed features that were typical of malignant epithelial cells. The STR genotyping analysis showed the ZJU-0430 cell line was of human origin and that it differed from other established cell lines. A mycoplasma examination confirmed ZJU-0430 to be free of mycoplasma. The PDT was approximately 19.81 h, which was shorter than GBC-SD cells. Taken together, these results suggest that ZJU-0430 cell line is a representative of the disease and may be used for pre-clinical investigation of the pathogenesis and treatment of GBC.

Recently, Devendra Chaudhary, et al. reported that 46.7% of GBCs have an abnormal karyotype [45]. In the present study, the karyotypes of ZJU-0430 cell line were found to be near-triploid both at primary and at P100, with the structural aberrations including gains, losses, and additional material of unknown origin on chromosomes 6 and 17 (one derivative chromosome 6 and two derivative chromosome 17) resulting from an unbalanced translocation with others chromosomes (chromosome 6 to an unknown chromosome, and chromosome 17 to the short arm of chromosome 5). An isochromosome was found for the short arm of chromosome 21; and two unknown marker chromosomes were also detected. None of these structural aberrations matched those in the previous cytogenetic studies of GBC [26, 37]. Cytogenetic abnormalities with mutations are usually related to oncogenic activation; therefore, further analysis may yield potential molecular targets in the context of this disease.

Mycoplasma can impede a whole range of cellular properties and functions and hinders the use of cells and their derived products in pharmacological area [46]. The culture supernatant derived from the ZJU-0430 cells was subjected to PCR analysis, and was found to be mycoplasma free. We have therefore identified the ZJU-0430 cell line as a useful tool to study GBC.

Although numerous GBC cell lines have been documented, including G-415, NOZ, KMG-A, GBK-1, FU-GBC-1, FU-GBC-2, PTHrP-GBK, GB-d1, TGBC1TKB, TGBC2TKB, OCUG-1, TUGBK-1, HAG-1, GBC-SD, SGC-996, EH-GB1, EH-GB2 and TJ-GBC2 [11–20, 22, 23, 25, 27, 37, 47, 48], the surface markers present in GBC have yet to characterized. Meanwhile, cancer stem cells have highly clinically relevant in the development, progression, aggressiveness, recurrence, and metastasis of tumours. Our study is the first to characterize progenitor cells in a newly established GBC cell line. Our results are consistent with other reports [49–51] and since we found these cells to be positive for CD24, CD44, CD29, and CD133, partially positive for CD184, and CD326, and negative for CD34, CD90, CD117, and CD338. Taken together, these data showed that ZJU-0430 cell had stemness properties.

GBC is highly aggressive in patients [52]; therefore, the malignant phenotype of ZJU-0430 cell line was explored both in vitro and in vivo. Our data showed that the migration, invasion, and proliferation abilities of ZJU-0430 are much greater than of GBC-SD cells, and in addition there were higher levels of expression of metastatic-related marker MMP-2. The cells were capable of forming solid tumours that were histologically identical to the original surgical specimen, indicating that the GBC cell line could be used as a reliable model for in vivo preclinical research.

An immunoblotting assay showed ZJU-0430 cells to be positive for biliary tract cancer epithelial marker (CK-pan, CK7, and CK19), which are found to be positive at high rates in real GBC specimens. The mesenchymal marker vimentin was also tested, but it is not found to be expressed. Since the patient (source of ZJU-0430) had been diagnosed with gastric carcinoma before GBC, gastric carcinoma markers were also explored on the xenograft tumour. The gastric carcinoma markers MUC2, MUC5AC, and MUC6 were expressed weakly or not at all. Since the colon and liver are contiguous with each other, the origin of ZJU-0430 cell was a little unclear. In this study, liver markers (AFP, Hepatocyte, Glypican-3, and GS) and colon markers (CK20, CAD17, CDX2, β-catenin, and SATB2) were used for staining of xenograft-derived tumours, with the result that all markers were either expressed only weakly, or were not express at all. In addition, the colon cancer marker CDX2 protein were also not found to be expressed on original GBC. Therefore, ZJU-0430 cell line was verified to be originated from the gallbladder primary site.

## Conclusion

We have successfully established a human GBC cell line referred to as ZJU-0430 that is identical to the original surgical specimen. This well-characterized GBC cell line should be helpful in the investigation of the pathogenesis of GBC, and may help in developing early diagnostic tools as well as potential targeted therapies.

## Additional files

**Additional file 1.** STR Profile Report for ZJU-0430 at P100.

**Additional file 2.** STR Profile Report for ZJU-0430 at P0.

**Additional file 3: Figure S1.** Immunophenotypic analysis for ZJU-0430 as determined by flow cytometry. The representative graph of CD24, CD44, CD29, CD34, CD90, CD117, CD133, CD184 CD326, CD338 protein expression were detected on primary cells and P100 cells of ZJU-0430.

**Additional file 4: Figure S2.** IHC for original gastric cancer sections. ZJU-0430 original gastric cancer tissues positively expressed most gastrointestinal tract markers (CK20, CAD17, and CDX2), and negative expressed β-catenin and SATB2.

**Additional file 5: Figure S3.** Characteristic of ZJU-0430 cell line in by scRNA-Seq data. (a) tSNE plot of ZJU-0430 cell line clusters defined by Seurat pipline before merging similar sub cell clusters. (b) UMAP plot of ZJU-0430 cell line clusters as showed in (a). (c) Dot heatmap of CD24, CD44, CD29, CD133 expression in each cell clusters. Heatmap showed the expression pattern of genes associated with Wnt signaling pathway (d), extracellular exosome (e), apoptotic signaling pathway (f) and nuclear transcribed mRNA catabolic process nonsense mediated (g).

## Data Availability

All data during this research are included in this published article.

## Abbreviations

STR: short tandem repeat
PDT: population doubling time
GBC: gallbladder carcinoma
CC: chronic cholecystitis
CT: computed tomography
RPMI: Roswell Park Memorial Institute
FBS: fetal bovine serum
DPBS: Dulbecco’s Phosphate Buffered Saline
BSA: bovine serum albumin fraction V
GEMs: gel beads in emulsions
UMI: unique molecular identifier
PCA: principal component analysis
ZUACUC: Zhejiang University Animal Care and Use Committee
PI: propidium, iodide
ATCC: American Type Culture Collection
H&E: haematoxylin and eosin
CST: cell signaling technology
DAB: 3,3′ diaminobenzidine tetrahydrochloride
