# Supplementary figures and images for: Characteristics of a novel cell line ZJU-0430 established from human gallbladder carcinoma
Source: Cancer Cell Int. 2019 Jul 22;19:190. doi: 10.1186/s12935-019-0911-1 (PMC6647153; doi:10.1186/s12935-019-0911-1)

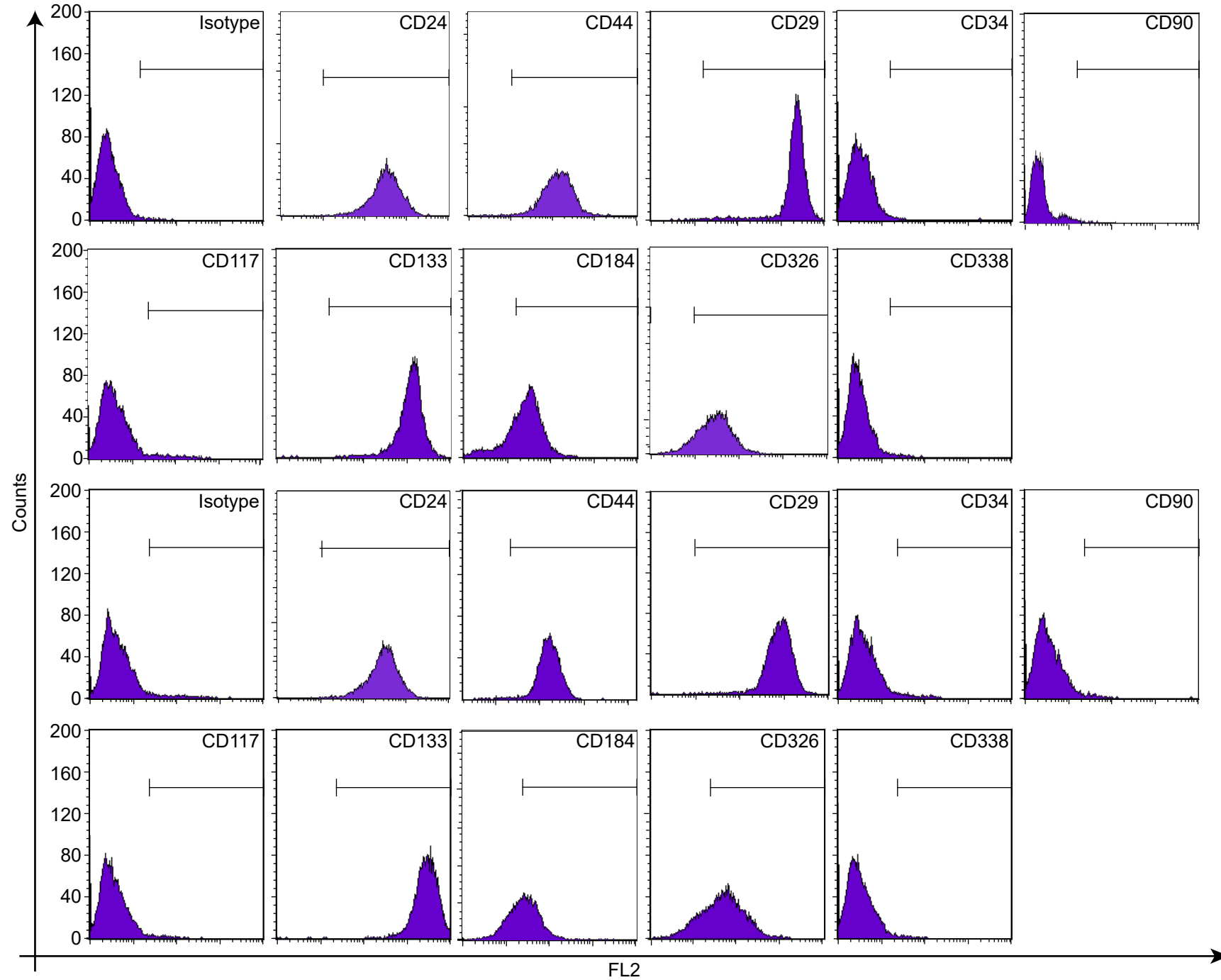

Supplement: Supplementary file 3 — Additional file 3: Figure S1. Immunophenotypic analysis for ZJU-0430 as determined by flow cytometry. The representative graph of CD24, CD44, CD29, CD34, CD90, CD117, CD133, CD184 CD326, CD338 protein expression were detected on primary cells and P100 cells of ZJU-0430. [file 12935_2019_911_MOESM3_ESM.pdf]

CK20

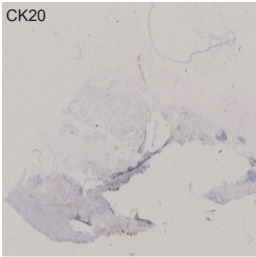

CAD17

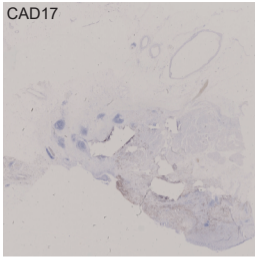

CDX2

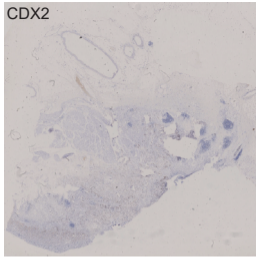

$\beta$ -catenin

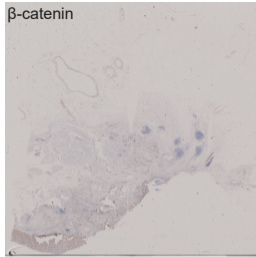

SATB2

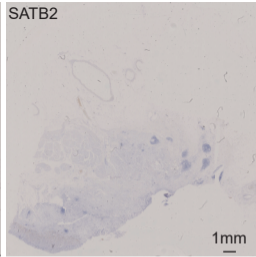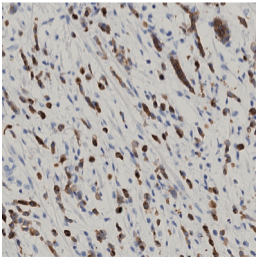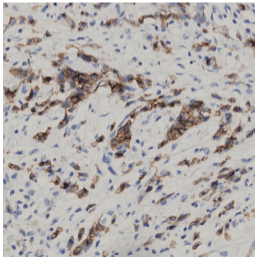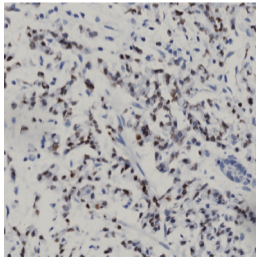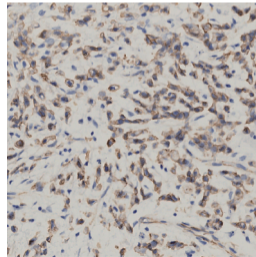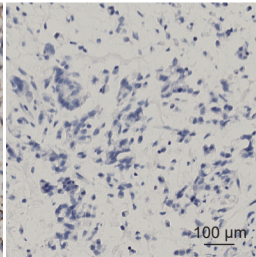

Supplement: Supplementary file 4 — Additional file 4: Figure S2. IHC for original gastric cancer sections. ZJU-0430 original gastric cancer tissues positively expressed most gastrointestinal tract markers (CK20, CAD17, and CDX2), and negative expressed β-catenin and SATB2. [file 12935_2019_911_MOESM4_ESM.pdf]
