# Supplementary material for: Characteristics of a novel cell line ZJU-0430 established from human gallbladder carcinoma
Source: Cancer Cell Int. 2019 Jul 22;19:190. doi: 10.1186/s12935-019-0911-1 (PMC6647153; doi:10.1186/s12935-019-0911-1)
Supplement: Supplementary file 5 — Additional file 5: Figure S3. Characteristic of ZJU-0430 cell line in by scRNA-Seq data. (a) tSNE plot of ZJU-0430 cell line clusters defined by Seurat pipline before merging similar sub cell clusters. (b) UMAP plot of ZJU-0430 cell line clusters as showed in (a). (c) Dot heatmap of CD24, CD44, CD29, CD133 expression in each cell clusters. Heatmap showed the expression pattern of genes associated with Wnt signaling pathway (d), extracellular exosome (e), apoptotic signaling pathway (f) and nuclear transcribed mRNA catabolic process nonsense mediated (g). [file 12935_2019_911_MOESM5_ESM.pdf]

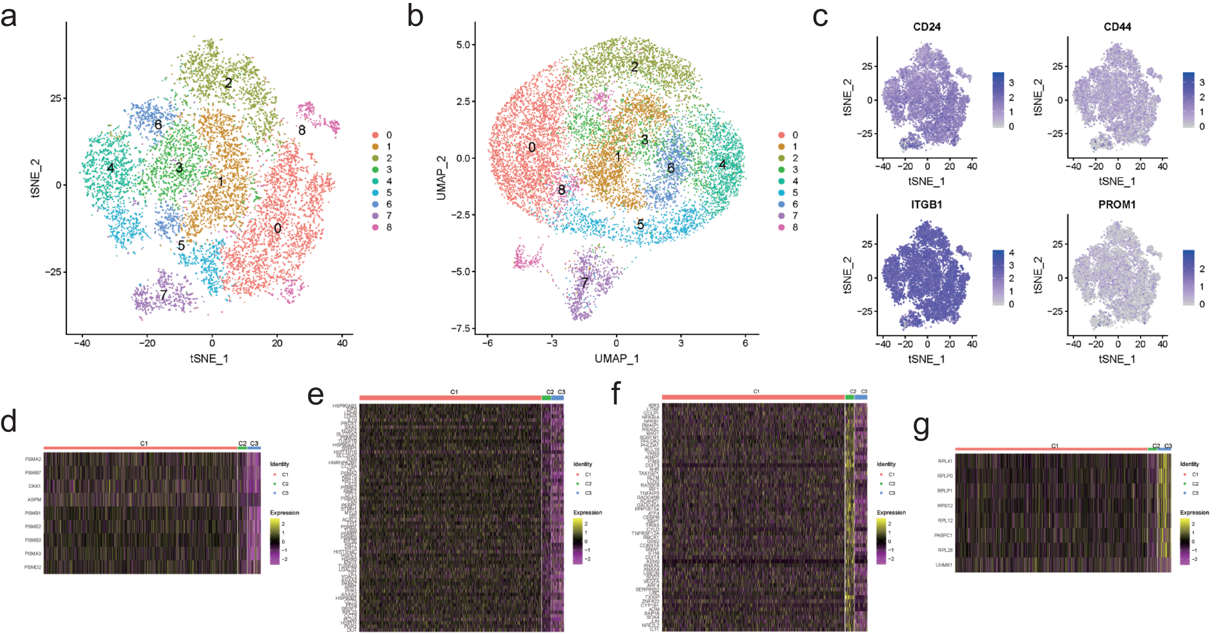
a
tSNE_2
tSNE_1
b
UMAP_2
UMAP_1
c
CD24
CD44
ITGB1
PROM1
d
e
f
g
C1
C2
C3
PSMA2
PSMB7
DKK1
ASPM
PSMB1
PSME2
PSMB3
PSMA3
PSMD2
RPL41
RPLP0
RPLP1
RPS12
RPL12
PABPC1
RPL28
UHMK1
Identity
Expression
